# Effectiveness of a long-lasting piperonyl butoxide-treated insecticidal net and indoor residual spray interventions, separately and together, against malaria transmitted by pyrethroid-resistant mosquitoes: a cluster, randomised controlled, two-by-two factorial design trial

**DOI:** 10.1016/S0140-6736(18)30427-6

**Published:** 2018-04-21

**Authors:** Natacha Protopopoff, Jacklin F Mosha, Eliud Lukole, Jacques D Charlwood, Alexandra Wright, Charles D Mwalimu, Alphaxard Manjurano, Franklin W Mosha, William Kisinza, Immo Kleinschmidt, Mark Rowland

**Affiliations:** a Department of Disease Control, London School of Hygiene & Tropical Medicine, London, UK; b MRC Tropical Epidemiology Group, London School of Hygiene & Tropical Medicine, London, UK; c National Institute for Medical Research, Mwanza Medical Research Centre, Mwanza, Tanzania; d Pan-African Malaria Vector Research Consortium, Kilimanjaro Christian Medical University College, Moshi, Tanzania; e Ministry of Health Community Development Gender Elderly and Children, National Malaria Control Program, Dar es Salaam, Tanzania; f National Institute for Medical Research, Amani Medical Research Centre, Muheza, Tanzania; g School of Pathology, Faculty of Health Sciences, University of Witwatersrand, Johannesburg, South Africa

## Abstract

**Background:**

Progress in malaria control is under threat by wide-scale insecticide resistance in malaria vectors. Two recent vector control products have been developed: a long-lasting insecticidal net that incorporates a synergist piperonyl butoxide (PBO) and a long-lasting indoor residual spraying formulation of the insecticide pirimiphos-methyl. We evaluated the effectiveness of PBO long-lasting insecticidal nets versus standard long-lasting insecticidal nets as single interventions and in combination with the indoor residual spraying of pirimiphos-methyl.

**Methods:**

We did a four-group cluster randomised controlled trial using a two-by-two factorial design of 48 clusters derived from 40 villages in Muleba (Kagera, Tanzania). We randomly assigned these clusters using restricted randomisation to four groups: standard long-lasting insecticidal nets, PBO long-lasting insecticidal nets, standard long-lasting insecticidal nets plus indoor residual spraying, or PBO long-lasting insecticidal nets plus indoor residual spraying. Both standard and PBO nets were distributed in 2015. Indoor residual spraying was applied only once in 2015. We masked the inhabitants of each cluster to the type of nets received, as well as field staff who took blood samples. Neither the investigators nor the participants were masked to indoor residual spraying. The primary outcome was the prevalence of malaria infection in children aged 6 months to 14 years assessed by cross-sectional surveys at 4, 9, 16, and 21 months after intervention. The endpoint for assessment of indoor residual spraying was 9 months and PBO long-lasting insecticidal nets was 21 months. This trial is registered with ClinicalTrials.gov, number NCT02288637.

**Findings:**

7184 (68·0%) of 10 560 households were selected for post-intervention survey, and 15 469 (89·0%) of 17 377 eligible children from the four surveys were included in the intention-to-treat analysis. Of the 878 households visited in the two indoor residual spraying groups, 827 (94%) had been sprayed. Reported use of long-lasting insecticidal nets, across all groups, was 15 341 (77·3%) of 19 852 residents after 1 year, decreasing to 12 503 (59·2%) of 21 105 in the second year. Malaria infection prevalence after 9 months was lower in the two groups that received PBO long-lasting insecticidal nets than in the two groups that received standard long-lasting insecticidal nets (531 [29%] of 1852 children *vs* 767 [42%] of 1809; odds ratio [OR] 0·37, 95% CI 0·21–0·65; p=0·0011). At the same timepoint, malaria prevalence in the two groups that received indoor residual spraying was lower than in groups that did not receive indoor residual spraying (508 [28%] of 1846 children *vs* 790 [44%] of 1815; OR 0·33, 95% CI 0·19–0·55; p<0·0001) and there was evidence of an interaction between PBO long-lasting insecticidal nets and indoor residual spraying (OR 2·43, 95% CI 1·19–4·97; p=0·0158), indicating redundancy when combined. The PBO long-lasting insecticidal net effect was sustained after 21 months with a lower malaria prevalence than the standard long-lasting insecticidal net (865 [45%] of 1930 children *vs* 1255 [62%] of 2034; OR 0·40, 0·20–0·81; p=0·0122).

**Interpretation:**

The PBO long-lasting insecticidal net and non-pyrethroid indoor residual spraying interventions showed improved control of malaria transmission compared with standard long-lasting insecticidal nets where pyrethroid resistance is prevalent and either intervention could be deployed to good effect. As a result, WHO has since recommended to increase coverage of PBO long-lasting insecticidal nets. Combining indoor residual spraying with pirimiphos-methyl and PBO long-lasting insecticidal nets provided no additional benefit compared with PBO long-lasting insecticidal nets alone or standard long-lasting insecticidal nets plus indoor residual spraying.

**Funding:**

UK Department for International Development, Medical Research Council, and Wellcome Trust.

**Research in context**

**Evidence before this study**

We did two searches in PubMed with no language restrictions or specified dates. In the first search on long-lasting insecticidal nets treated with piperonyl butoxide (PBO), we used the search terms “malaria” and “long lasting insecticidal net” in combination with “piperonyl butoxide”, “Olyset Plus”, or “PermaNet 3.0”, which produced no references that were randomised controlled trials (RCTs). In a 2015 review of PBO long-lasting insecticidal nets, WHO concluded that although they appeared to have an increased efficacy the evidence was too inadequate to justify a switch from pyrethroid only to PBO nets across all settings. Because of the potential for an antagonistic effect between PBO and organophosphates, WHO also recommended that PBO nets should not be used in areas programmed for indoor residual spraying with pirimiphos-methyl capsule suspension.

In the second search on combined vector control interventions, we included the search term “malaria” with one or more of the following: “long lasting insecticidal net” or “insecticide treated net”, “indoor residual spraying”, “vector control”, “pirimiphos methyl”, and “combined interventions”. We identified three other RCTs that have examined combined intervention of long-lasting insecticidal nets and indoor residual spraying. The Gambian RCT, which used DDT (dichlorodiphenyltrichloroethane) as the indoor residual spraying component showed no additional protection compared with long-lasting insecticidal nets alone. This finding could be explained by the high usage of nets or the properties of the sprayed insecticide used. In Benin, there was no advantage to combining indoor residual spraying and long-lasting insecticidal nets; however, suboptimal coverage of nets and the short residual effect of the spray used (bendiocarb) might have affected the effectiveness of the combination intervention. In an earlier Tanzanian study, where coverage of long-lasting insecticidal nets was moderate and pyrethroid resistance present, two rounds of indoor residual spraying with bendiocarb combined with long-lasting insecticidal nets were more effective than long-lasting insecticidal nets alone.

**Added value of this study**

Our study is the first RCT to report that PBO-treated long-lasting insecticidal nets were more effective than standard pyrethroid long-lasting insecticidal nets against malaria infection and transmission. It also provides the strongest evidence to date of the negative effect of high-level pyrethroid resistance on the use and efficacy of standard nets. This study is also the first RCT to provide evidence for the effect of long-term malaria control of the first long-lasting organophosphate formulation to be developed specifically for indoor residual spraying. The RCT provides new evidence on the added value and risks of combining indoor residual spraying and long-lasting insecticidal nets, particularly PBO nets.

**Implications of all the available evidence**

This study provides justification for the increase in deployment and use of PBO long-lasting insecticidal nets. As a direct consequence of this trial, WHO revised its policy on long-lasting insecticidal nets in September, 2017, gave interim endorsement to pyrethroid-PBO nets as a new WHO class of vector control product, and recommended that PBO nets be deployed for prevention of malaria where vectors are resistant to pyrethroids provided that vector control coverage is not compromised. This endorsement would include many endemic areas in Africa where standard long-lasting insecticidal nets are currently used. The demonstration that long-lasting insecticidal nets with an appropriate change of active ingredient can continue to tackle transmission by pyrethroid-resistant vector populations will ensure the viability of this approach as well as justifies the continued investment and search for alternative insecticides for use on nets.

Finally, the organophosphate indoor residual spraying formulation is the first long-lasting, non-pyrethroid insecticide to provide malaria control for at least 9 months over two transmission seasons in the same year. This finding justifies the scale up and use of indoor residual spraying in sub-Saharan Africa and the 12-year investment into long-lasting alternatives to pyrethroid and DDT for indoor spraying between private and public sector organisations.

## Introduction

Long-lasting insecticidal nets and indoor residual spraying are the cornerstones of malaria control in sub-Saharan Africa. Together with effective treatment, these interventions are estimated to have globally reduced malaria morbidity by 41% and mortality by 62% between 2000 and 2015.^1^ Despite this public health success, recent wide-scale selection of insecticide resistance in the mosquito vectors across Africa threatens to reverse the present gains.^2^ Development and evaluation of new strategies and tools are needed to address the threat of resistance and will accelerate progress towards elimination.

The range of insecticides available for indoor residual spraying is limited. For long-lasting insecticidal nets, the range is particularly restricted because pyrethroids are the only class of insecticides recommended by WHO for nets. Evidence from indoor residual spraying programmes suggests that pyrethroid resistance can contribute to operational control failure—eg, in South Africa, control was only restored once the pyrethroid was replaced by an insecticide to which vectors were susceptible.^3^ By contrast, the negative effect of pyrethroid resistance on the effectiveness of long-lasting insecticidal nets has been less clear and harder to quantify than indoor residual spraying.^4^ Although entomological evidence suggests that these nets are becoming less effective at killing mosquitoes in household conditions when resistance develops,^5^, ^6^ the physical barrier provided by the net, especially when new and intact, might mitigate some of the loss in bioefficacy due to resistance.^7^ Cohort studies have shown that long-lasting insecticidal nets remain protective against malaria infection in areas of moderate insecticide resistance in Malawi^8^ and Kenya,^9^ whereas no reduction in incidence was observed after the distribution of these nets in Uganda.^10^

Anticipating the possible failure of current control tools due to resistance, WHO has encouraged the industry to develop new types of long-lasting insecticidal nets and new insecticides for indoor residual spraying. One of these developments is a new long-lasting insecticidal net that uses piperonyl butoxide (PBO). PBO is a chemical synergist that acts by inhibiting enzymes involved in the natural defense mechanisms of insects, which results in pyrethroid not being detoxified in the insect and the pyrethroid on the long-lasting insecticidal net remaining potent against mosquitoes despite resistance. Such PBO-pyrethroid-treated long-lasting insecticidal nets appear to have similar or better efficacy against resistant mosquitoes under controlled household conditions than standard long-lasting insecticidal nets that do not have PBO.^11^, ^12^ In September, 2015, a WHO expert group reviewed the evidence for PBO long-lasting insecticidal nets to define their deployment. Despite awaiting for more conclusive evidence from community randomised controlled trials (RCTs) with epidemiological outcomes, WHO, nevertheless, has recommended a small rollout in specific situations.^13^

Although the range of insecticide classes suitable for indoor residual spraying use is wider than long-lasting insecticidal nets, few insecticides are effective for more than a few months when sprayed onto walls and this limitation has been a constraint on their adoption and use. The organophosphate pirimiphos-methyl is an exception, and the recently developed long-lasting formulation, Actellic 300CS (Syngenta, Switzerland), is effective for up to 10 months when used for indoor residual spraying.^14^ It is now being deployed in several African countries instead of carbamates.^15^

In attempts to accelerate malaria control progress, long-lasting insecticidal nets and indoor residual spraying have been deployed together in several countries.^1^ The advantage of combined intervention has, however, been the focus of considerable debate because both observational and RCTs have produced contradictory evidence. In The Gambia and Benin, no difference in malarial outcomes were reported when both control strategies were deployed together compared with long-lasting insecticidal nets alone,^16^, ^17^ whereas in Tanzania an increased effectiveness was observed when they were used in combination.^18^ On the basis of these data, the effect observed would seem to depend on the insecticide combination used, the vectors present, the coverage and quality of the intervention, and the level and type of insecticide resistance in the vectors.

To develop an improved strategy for control of malaria transmitted by pyrethroid-resistant mosquito vectors, we aimed to compare the effectiveness of PBO long-lasting insecticidal nets with standard long-lasting insecticidal nets as single interventions and in combination with the long-lasting indoor residual spraying of pirimiphos-methyl.

## Methods

### Study design and participants

**Figure 1:**
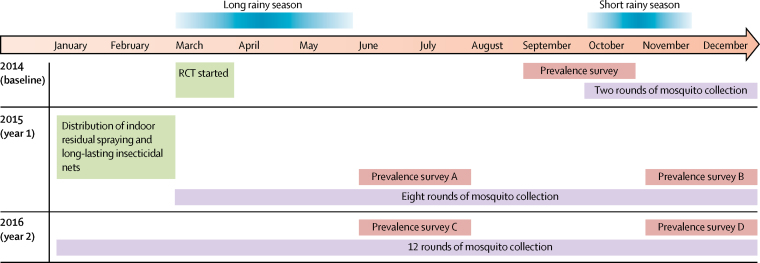
Study timetable RCT=randomised controlled trial.

We did a cluster RCT of four groups using a two-by-two factorial design. The RCT started on March 1, 2014. The post-intervention assessment period was initially planned for 18 months (from Jan 1, 2015, to June 30, 2016) and was subsequently extended on our request to the funding agency to 24 months (from Jan 1, 2014, to Dec 31, 2016) to enable further assessment of the PBO long-lasting insecticidal net (figure 1).

The study area was Muleba district of the Kagera region in northwest Tanzania, and comprised 40 villages. In 2011, malaria infection prevalence in children was 23%.^18^ *Anopheles gambiae* and *Anopheles arabiensis* were the only vectors found in 2012. High levels of resistance to pyrethroids have been reported in *A gambiae* in the study area, and synergy bioassay tests done with PBO and pyrethroid together partially restored the toxicity of pyrethroids.^19^ All villages and hamlets with malaria prevalence more than 20% in 2011 were eligible for inclusion in the present trial. Our trial comprised 48 clusters, each divided into an inner core area, which was used for the measurement of study outcomes, and an outer buffer zone of at least 300 m to reduce spill-over effects between clusters.^20^ Core and buffer areas of each cluster received the same intervention. All households in the core area with children aged 6 months to 14 years were eligible for malaria cross-sectional survey and mosquito surveillance. We excluded children who were severely ill. Village meetings were held with village leaders, hamlet representatives, community health agents, and villagers to inform them about the trial.

The trial was approved by the ethics review committees of the Kilimanjaro Christian Medical University College, the London School of Hygiene & Tropical Medicine, and the Tanzanian Medical Research Coordinating Committee (NIMR/HQ/R.8a/VolIX/1803). A trial steering committee reviewed progress. Written informed consent from parents or guardians was obtained for each survey and entomology collection.

### Randomisation and masking

We used restricted randomisation to allocate the 48 clusters to the four study groups: standard long-lasting insecticidal nets, PBO long-lasting insecticidal nets, standard long-lasting insecticidal nets plus indoor residual spraying, and PBO long-lasting insecticidal nets plus indoor residual spraying. We limited potential imbalance using three restriction variables: malaria infection prevalence in children aged 6 months to 14 years, usage of long-lasting insecticidal nets, and socioeconomic status, as recorded in the baseline survey between September and October, 2014. Of the 200 000 random allocations, 29 478 met the restriction criteria of no more than 7% difference in mean malaria prevalence, 10% in mean usage of long-lasting insecticidal nets, and 10% of households in the lowest socioeconomic status tertile between study groups. After verifying that clusters were independently allocated to study groups, we randomly chose one of the eligible allocations.

We masked the inhabitants of each cluster to the type of long-lasting insecticidal nets received. The two types of nets were of similar colour and shape, and only distinguishable by label codes and coloured thread inserted during manufacture. Additionally, we masked field staff, who took blood samples in the cross-sectional surveys, to the study groups the clusters were assigned to. It was not possible to mask either the investigators or the participants to the treatment allocation of indoor residual spraying.

### Procedures

We used the following vector control products: Olyset Net (Sumitomo Chemicals, Japan) containing 2% permethrin (standard long-lasting insecticidal net), Olyset Plus (Sumitomo Chemicals, Japan) containing 2% permethrin and 1% PBO (PBO long-lasting insecticidal net), and Actellic 300CS containing microencapsulated pirimiphos-methyl (indoor residual spraying).

We georeferenced all houses in the study using hand-held global positioning system units (Legend eTrex, Garmin, USA). The indoor residual spraying campaign was done once only in February, 2015, by the Research Triangle Institute funded by the President's Malaria Initiative. In the two groups assigned to indoor residual spraying intervention, Actellic 300CS was sprayed to the interior walls and ceilings of each dwelling at the recommended dosage of 1 g/m^2^. The residual decay of Actellic 300CS was monitored by a laboratory technician every 3 months on representative wall surfaces in several houses using WHO Cone bioassay tests (Universiti Sains Malaysia, Malaysia) and a reference strain of susceptible *A gambiae*. The permethrin and PBO contents of the long-lasting insecticidal nets were determined by high-performance liquid chromatography at yearly intervals for 2 years.

Distribution of long-lasting insecticidal nets and health education communication on net usage were done in each cluster by the Tanzania Communication and Development Centre. On the basis of census data, each household received one net per two people. Altogether, 45 000 standard long-lasting insecticidal nets and 45 000 PBO long-lasting insecticidal nets were distributed in February, 2015. Nets already owned were not removed but householders were requested to use the study nets provided.

Cross-sectional household and malaria infection prevalence surveys were done by project field assistants and nurses at baseline in September and October, 2014, and after intervention at the end of each malaria transmission season (June to July and November to December) in 2015 and 2016 (figure 1). During each survey, we randomly sampled 55 households with children aged 6 months to 14 years from the core area of each cluster using the census lists. We then selected up to three eligible children per house at random and recorded information about the number of residents, household assets, house structure, educational status, and use of malaria preventive measures (long-lasting insecticidal nets or other). The minimum target was 80 children per cluster. Enrolled children reported to the clinical team the next day and were tested for malaria using a rapid diagnostic test (CareStart Malaria HRP2/pLDH(pf/PAN) Combo, DiaSys, UK) and for haemoglobin concentration using HemoCue Hb 201+ (HemoCue AB, Sweden). Children diagnosed as malaria positive by the rapid diagnostic test were treated with artemether-lumefantrine according to national guidelines. Any child presenting with illness during the surveys was treated or referred to the nearest health facility if symptoms were severe.

Mosquito surveillance was done from March, 2015, to December, 2016, in each cluster by a project field assistant for one night per month in seven randomly selected houses per cluster using CDC Miniature Light Trap Model 512 (John W Hock Company, USA) as a proxy for human biting rates.^17^ We morphologically identified the collected anophelines to species level^21^ and tested a subsample for *Plasmodium falciparum* circumsporozoite protein.^22^ PCR TaqMan assay^23^ was used to distinguish the two sibling species (*A gambiae* and *A arabiensis*) and to identify mutation in the voltage-gated sodium channel (Vgsc-1014F and Vgsc-1014S) associated with resistance to pyrethroids.^24^ Using wild caught *A gambiae* and *Anopheles funestus* of unknown age, the frequency of pyrethroid resistance was determined using 0·75% permethrin papers in WHO cylinder tests. We determined resistance intensity using CDC bottle bioassays and probit analysis to estimate the ratio of the permethrin concentration needed to kill 50% of wild mosquitoes relative to the susceptible strain.

### Outcomes

The primary outcome was the prevalence of *Plasmodium* spp infection measured by the rapid diagnostic test in children aged 6 months to 14 years assessed by the cross-sectional surveys. The trial was initially funded for 18 months after intervention. Although this period could have been chosen as the endpoint, it was not known for how long the PBO and pyrethroid active ingredients in the long-lasting insecticidal nets would last. This effect needed to be monitored every transmission season. We subsequently secured extension from the funding agency for 24 months. WHO then reset the policy agenda declaring that new types of long-lasting insecticidal nets (such as the PBO net) should be effective for at least two transmission seasons or 2 years. The primary endpoint for the indoor residual spraying was 1 year, based on reports of duration of residual activity. Because the two intervention products were being assessed separately and in combination, the main endpoint for assessment of the indoor residual spraying was 9 months and the PBO long-lasting insecticidal nets was 9 months and then 21 months.

The secondary main outcome was malaria transmission or entomological inoculation rate, defined as the mean number of infective mosquito bites per household per month, during the first year and second year after intervention. Other secondary endpoints were the proportion of children with moderate-to-severe anaemia (defined as haemoglobin <8 g/dL), the sporozoite rate (the proportion of anopheline mosquitoes collected that were infected with malaria sporozoites), and anopheline population density.

### Statistical analysis

This study had 80% power^25^ to detect a relative reduction in prevalence of infection of at least 28% (prevalence ratio 0·72) for each of the two main effects (ie, indoor residual spraying *vs* no indoor residual spraying, and PBO long-lasting insecticidal nets *vs* no PBO long-lasting insecticidal nets) and a 40% difference between any of the individual groups, with 24 clusters of 80 individuals per cluster being tested in each of these comparisons, and assuming a mean prevalence of 20% in the reference groups and a coefficient of variation of 0·3 (based on data from the earlier study).^18^

Statistical analysis was done using Stata (version 12). All statistical inferences allowed for within-cluster correlation of responses by use of a robust variance estimator to calculate SEs. No allowance was made for multiplicity of testing in the analyses. In the intention-to-treat analysis, logistic regression was used to estimate odds ratios (ORs) of the effect of each of the two interventions (PBO long-lasting insecticidal nets *vs* standard long-lasting insecticidal nets, and indoor residual spraying *vs* no indoor residual spraying) on prevalence of infection and prevalence of anaemia. We estimated interaction between the two main effects by including an appropriate term in the model. We also examined the effect of each intervention (PBO long-lasting insecticidal nets, combination of standard long-lasting insecticidal nets plus indoor residual spraying, and combination of PBO long-lasting insecticidal nets plus indoor residual spraying) compared with the control group (standard long-lasting insecticidal net). Effects were interpreted in relation to a postulated minimum difference of 28% for factorial analysis and 40% for the analysis of each intervention. Analysis of anaemia was restricted to children aged 6 months to 4 years. The per-protocol analysis is available in the appendix.

Vector density and entomological inoculation rate were analysed with negative binomial regression, after adjusting for baseline. Entomological inoculation rate was estimated as the mean number of sporozoite-infected Anopheles per house per night^26^ and weighted to account for the proportion of collected Anopheles processed for sporozoites. The proportion of sporozoite-infected mosquitoes (the sporozoite rate) was compared using logistic regression.

This trial is registered with ClinicalTrials.gov, number NCT02288637.

### Role of the funding source

The funder of the study had no role in the study design, data collection, data analysis, data interpretation, or writing of the report. The corresponding author had full access to all the data in the study and had final responsibility for the decision to submit for publication.

## Results

**Figure 2:**
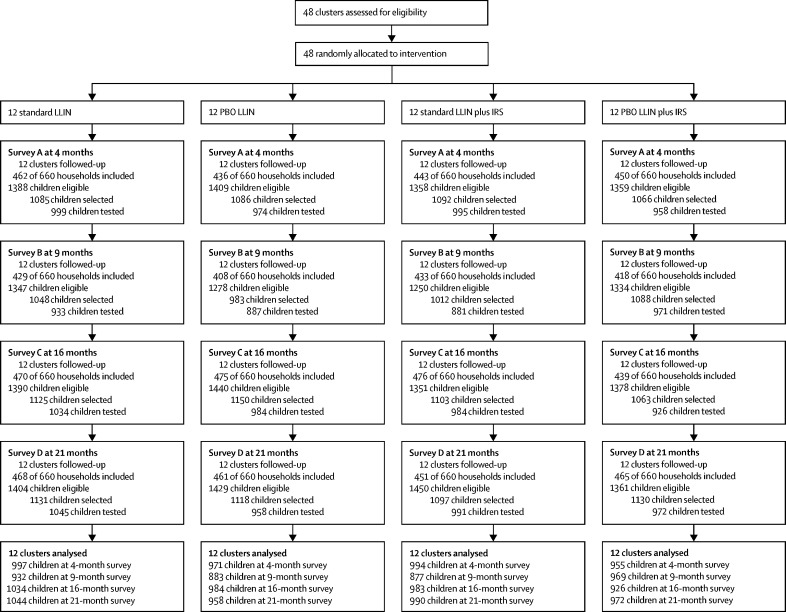
Trial profile LLIN=long-lasting insecticidal net. PBO=piperonyl butoxide. IRS=indoor residual spraying.

The study area comprised 29 365 households and a population of 135 900. Of the 10 560 households selected for post-intervention survey, 7184 (68·0%) were included whereas 1127 (10·7%) were ineligible (no children younger than 15 years), 150 (1·4%) refused, 1543 (14·6%) were absent, and 556 (5·3%) were unvisited. Of the 17 377 eligible children selected, 15 492 (89·2%) attended for testing (figure 2). Pre-intervention household and demographic characteristics, as well as coverage and usage of long-lasting insecticidal nets were similar between study groups (table 1). Malaria infection prevalence was reported in 2499 (65%) of 3861 children at baseline, and any difference between groups were within the tolerances set for the constrained randomisation. The average indoor Anopheles density was 27·6 per house per night and the proportion of mosquitoes with sporozoites was 4·5%. Of the 13 689 Anopheline mosquitoes collected, 13 106 (95·7%) were *A gambiae* sensu lato and 510 (3·7%) were *A funestus*. Of the 990 *A gambiae* sensu lato identified to species, 946 (95·6%) were *A gambiae* sensu stricto and 44 (4·4%) were *A arabiensis*.

**Table 1:** Baseline characteristics

	**Standard LLIN**	**PBO LLIN**	**Standard LLIN plus IRS**	**PBO LLIN plus IRS**
**Study cluster characteristics**				
Total population in core and buffer areas	33 820	32 861	38 081	31 138
Population in core area	15 947	16 282	16 358	14 845
**Household characteristics**				
Median altitude of the households selected (range; N)	1330 (1138–1654; 465)	1275 (1138–1563; 500)	1298 (1129–1486; 508)	1338 (1152–1543; 510)
Households in the lowest socioeconomic category	146/464 (31%)	166/534 (31%)	198/528 (38%)	163/467 (35%)
Households with adequate long-lasting insecticidal nets	174/545 (32%)	223/582 (38%)	230/580 (40%)	211/561 (38%)
Households with ≥1 long-lasting insecticidal nets	356/545 (65%)	410/582 (70%)	402/581 (69%)	378/561 (67%)
Long-lasting insecticidal nets use in all age groups	902/2996 (30%)	810/3078 (26%)	882/3197 (28%)	810/3078 (26%)
**Children characteristics**				
Median age, years (IQR; N)	6 (3–10; 885)	6 (3–9; 991)	6 (3–10; 1017)	6 (3–10; 967)
Long-lasting insecticidal net use in selected children	348/891 (39%)	315/992 (32%)	315/1018 (31%)	307/970 (32%)
Malaria infection prevalence	600/885 (68%)	606/991 (61%)	678/1018 (67%)	615/967 (64%)
Anaemia prevalence in children <5 years*	36/328 (11%)	36/378 (10%)	34/372 (9%)	29/362 (8%)
Median haemoglobin concentration in children <5 years, g/dL (IQR; N)	10·4 (9·2–11·5; 328)	10·6 (9·1–11·7; 378)	10·6 (9·2–11·7; 372)	10·6 (9·6–11·6; 362)
**Entomological characteristics**				
Mean number of vectors found indoors per house per night (95% CI; N)	17·0 (0–34·7; 129)	37·0 (4·0–70·1; 119)	11·8 (0–24·7; 117)	43·6 (9·7–77·6; 129)
Sporozoite rate	39/809 (5%)	59/1085 (5%)	37/733 (5%)	35/1161 (3%)

Data are n/N (%), unless stated otherwise. Data for household, children, and entomological characteristics are only for the core area. LLIN=long-lasting insecticidal net. PBO=piperonyl butoxide. IRS=indoor residual spraying.

* Anaemia was clinically diagnosed as <8 g/dL.

Between baseline and the first cross-sectional survey 4 months after intervention, long-lasting insecticidal net ownership (≥one net per household) increased to 1690 (97·6%) of 1732 households, access (household with enough long-lasting insecticidal net per sleeping place) increased to 1550 (89·6%) of 1730, and long-lasting insecticidal net use increased to 7807 (76·9%) of 10 152 (appendix). Long-lasting insecticidal net usage was similar between groups and between surveys during the first year. In the second year, 21 months after intervention, access decreased to 1291 (70·2%) of 1839 households and usage to 5905 (56·0%) of 10 551 residents. Most long-lasting insecticidal nets observed were those deployed from this study. In the standard long-lasting insecticidal net (Olyset Net), permethrin concentration at 0 months of use was 21·4 g/kg and 21·5 g/kg after 12 months of use and decreased to 16·7 g/kg after 21 months of use. For the PBO long-lasting insecticidal net (Olyset Plus), permethrin concentration at 0 months of use was 20·9 g/kg, which decreased to 14·7 g/kg after 12 months and to 12·2 g/kg after 21 months, while PBO concentration on Olyset Plus also decreased from 9·5 g/kg at 0 months to 2·9 g/kg after 12 months and to 1·6 g/kg after 21 months of use.

827 (94%) of 878 households selected for the survey received indoor residual spraying in the two groups assigned to this intervention. The insecticide residues on sprayed walls decayed gradually over the year; mosquito mortality in WHO cone bioassays was 99% (566 of 570 exposed mosquitoes died, 95% CI 97·9–100) shortly after spraying, 82% (356 of 432, 75·4–89·5) after 9 months, and 59% (495 of 840, 51·4–66·4) after 12 months.

In the intention-to-treat factorial analysis for the prevalence of malaria infection, the effect of indoor residual spraying versus no indoor residual spraying was evident at 4 months (OR 0·50, 95% CI 0·31–0·82; p=0·0071) whereas there was no evidence of a difference between PBO long-lasting insecticidal nets and standard long-lasting insecticidal nets (0·68, 0·39–1·18; p=0·1630; table 2). A clear effect was observed 9 months after intervention for indoor residual spraying versus no indoor spraying (OR 0·33, 95% CI 0·19–0·55; p<0·0001) and PBO long-lasting insecticidal nets versus standard nets (OR 0·37, 0·21–0·65; p=0·0011). During the second year, the prevalence of malaria infection in the PBO long-lasting insecticidal net groups remained less than in the standard long-lasting insecticidal net groups (OR 0·47, 95% CI 0·26–0·87; p=0·0173 after 16 months of intervention; and 0·40, 0·20–0·81; p=0·0122 after 21 months of intervention), whereas the effect of the single round of indoor residual spraying applied previously waned over time (OR 0·59, 95% CI 0·34–1·04 after 16 months; and 0·58, 0·29–1·14 after 21 months; table 2). The only significant interaction was at 9 months (OR 2·43, 95% CI 1·19–4·97; p=0·0158), suggesting that at this point in time the combined effect of indoor residual spraying and PBO long-lasting insecticidal net was less than the additive effect of each of the two effects alone.

**Table 2:** ITT analysis of malaria infection prevalence by main effect and for each individual intervention at 4, 9, 16, and 21 months after intervention

		**n/N (%)**	**OR (95% CI)**	**p value**
**Survey A, 2015, 4 months after intervention**				
Main effect comparison (factorial analysis)				
	No PBO LLIN*	936/1991 (47%)	1 (ref)	..
	PBO LLIN†	798/1926 (41%)	0·68‡ (0·39–1·18)	0·1630
	No IRS§	998/1968 (51%)	1 (ref)	..
	IRS¶	736/1949 (38%)	0·50‡ (0·31–0·82)	0·0071
	Interaction coefficient	..	1·37 (0·66–2·86)	0·3825
Individual group comparison				
	Standard LLIN	553/997 (55%)	1 (ref)	..
	PBO LLIN	445/971 (46%)	0·68‖ (0·39–1·18)	0·1630
	Standard LLIN plus IRS	383/994 (39%)	0·50‖ (0·31–0·82)	0·0071
	PBO LLIN plus IRS	353/955 (37%)	0·47‖ (0·28–0·79)	0·0048
**Survey B, 2015, 9 months after intervention**				
Main effect comparison (factorial analysis)				
	No PBO LLIN*	767/1809 (42%)	1 (ref)	..
	PBO LLIN†	531/1852 (29%)	0·37 (0·21–0·65)	0·0011
	No IRS§	790/1815 (44%)	1 (ref)	..
	IRS¶	508/1846 (28%)	0·33 (0·19–0·55)	<0·0001
	Interaction coefficient	..	2·43 (1·19–4·97)	0·0158
Individual group comparison				
	Standard LLIN	515/932 (55%)	1 (ref)	..
	PBO LLIN	275/883 (31%)	0·37 (0·21–0·65)	0·0011
	Standard LLIN plus IRS	252/877 (29%)	0·33 (0·19–0·55)	<0·0001
	PBO LLIN plus IRS	256/969 (26%)	0·29 (0·17–0·49)	0·0001
**Survey C, 2016, 16 months after intervention**				
Main effect comparison (factorial analysis)				
	No PBO LLIN*	941/2017 (47%)	1 (ref)	..
	PBO LLIN†	611/1910 (32%)	0·47 (0·26–0·87)	0·0173
	No IRS§	890/2018 (44%)	1 (ref)	..
	IRS¶	662/1909 (35%)	0·59‡ (0·34–1·04)	0·0652
	Interaction coefficient	..	1·30 (0·59-2·86)	0·5045
Individual group comparison				
	Standard LLIN	548/1034 (53%)	1 (ref)	..
	PBO LLIN	342/984 (35%)	0·47‖ (0·26–0·87)	0·0173
	Standard LLIN plus IRS	393/983 (40%)	0·59‖ (0·34–1·04)	0·0652
	PBO LLIN plus IRS	269/926 (29%)	0·36 (0·20–0·66)	0·0014
**Survey D, 2016, 21 months after intervention**				
Main effect comparison (factorial analysis)				
	No PBO LLIN*	1255/2034 (62%)	1 (ref)	..
	PBO LLIN†	865/1930 (45%)	0·40‡ (0·20–0·81)	0·0122
	No IRS§	1150/2002 (57%)	1 (ref)	..
	IRS¶	970/1962 (49%)	0·58‡ (0·29–1·14)	0·1130
	Interaction coefficient	..	1·59 (0·62–4·07)	0·3282
Individual group comparison				
	Standard LLIN	710/1044 (68%)	1 (ref)	..
	PBO LLIN	440/958 (46%)	0·40‖ (0·20–0·81)	0·0122
	Standard LLIN plus IRS	545/990 (55%)	0·58‖ (0·29–1·14)	0·1130
	PBO LLIN plus IRS	425/972 (44%)	0·37‖ (0·19–0·73)	0·0056

ORs for the factorial analysis compared the two main intervention effects (no PBO LLIN *vs* PBO LLIN, and no IRS *vs* IRS) and their interaction, and compared each of the intervention to the standard LLIN in the individual group analysis. OR was unadjusted for baseline plasmodium infection prevalence. Plasmodium infection prevalence is reported for children aged 6 months to 14 years. OR=odds ratio. LLIN=long-lasting insecticidal net. PBO=piperonyl butoxide. IRS=indoor residual spraying. ITT=intention to treat.

* Standard LLIN and standard LLIN plus IRS.

† PBO LLIN and PBO LLIN plus IRS.

‡ Reduction in prevalence is less than the 28% difference defined a priori for the main effect.

§ Standard LLIN and PBO LLIN.

¶ Standard LLIN plus IRS and PBO LLIN plus IRS.

‖ Reduction in prevalence is less than the 40% defined a priori for the individual arm comparison.

In the analysis of the individual group comparisons, the difference in malaria infection prevalence between the reference group (standard long-lasting insecticidal net) and the PBO long-lasting insecticidal net group or the combination of the standard net plus indoor residual spraying group was greater than that observed in the factorial analysis at every timepoint between PBO nets and non-PBO nets or between indoor residual spraying and no indoor spraying (table 2). The individual group comparison also provides information about the effect of the PBO long-lasting insecticidal net plus indoor residual spraying intervention.

Prevalence of severe-to-moderate anaemia was lower for the groups receiving PBO long-lasting insecticidal net compared with their standard long-lasting insecticidal net reference groups, and was also lower in the groups receiving indoor residual spraying than in the non-indoor residual spraying reference groups in the surveys after 9 months and 16 months intervention (table 3). Results of the per-protocol analyses of malaria infection and anaemia were similar to that of the intention-to-treat analyses (appendix).

**Table 3:** ITT analysis of anaemia prevalence by main effect and for each individual intervention at 4, 9, 16 and 21 months after intervention

		**n/N (%)**	**OR (95% CI)**	**p value**
**Survey A, 2015, 4 months after intervention**				
Main effect comparison (factorial analysis)				
	No PBO LLIN*	28/664 (4%)	1 (ref)	..
	PBO LLIN†	15/675 (2%)	0·39 (0·11–1·30)	0·1221
	No IRS‡	23/665 (3%)	1 (ref)	..
	IRS§	20/674 (3%)	0·69 (0·24–1·98)	0·4792
	Interaction coefficient	..	1·75 (0·37–8·14)	0·4696
Individual group comparison				
	Standard LLIN	16/320 (5%)	1 (ref)	..
	PBO LLIN	7/345 (2%)	0·39 (0·11–1·30)	0·1221
	Standard LLIN plus IRS	12/344 (3%)	0·69 (0·24–1·98)	0·4792
	PBO LLIN plus IRS	8/330 (2%)	0·47 (0·18–1·25)	0·1268
**Survey B, 2015, 9 months after intervention**				
Main effect comparison (factorial analysis)				
	No PBO LLIN*	20/580 (3%)	1 (ref)	..
	PBO LLIN†	13/603 (2%)	0·31 (0·11–0·88)	0·0292
	No IRS‡	23/584 (4%)	1 (ref)	..
	IRS§	10/599 (2%)	0·16 (0·04–0·69)	0·0149
	Interaction coefficient	..	7·51 (1·09–51·69)	0·0408
Individual group comparison				
	Standard LLIN	17/281 (6%)	1 (ref)	..
	PBO LLIN	6/303 (2%)	0·31 (0·11–0·88)	0·0292
	Standard LLIN plus IRS	3/299 (1%)	0·16 (0·04–0·69)	0·0149
	PBO LLIN plus IRS	7/300 (2%)	0·37 (0·11–1·22)	0·1004
**Survey C, 2016, 16 months after intervention**				
Main effect comparison (factorial analysis)				
	No PBO LLIN*	20/577 (3%)	1 (ref)	..
	PBO LLIN†	7/574 (1%)	0·23 (0·08–0·65)	0·0068
	No IRS‡	20/571 (4%)	1 (ref)	..
	IRS§	7/580 (1%)	0·22 (0·08–0·64)	0·0064
	Interaction coefficient	..	3·46 (0·48–24·80)	0·2108
Individual group comparison				
	Standard LLIN	16/279 (6%)	1 (ref)	..
	PBO LLIN	4/292 (1%)	0·23 (0·08–0·65)	0·0068
	Standard LLIN plus IRS	4/298 (1%)	0·22 (0·08–0·64)	0·0064
	PBO LLIN plus IRS	3/282 (1%)	0·18 (0·05–0·84)	0·0301
**Survey D, 2016, 21 months after intervention**				
Main effect comparison (factorial analysis)				
	No PBO LLIN*	19/586 (3%)	1 (ref)	..
	PBO LLIN†	24/582 (4%)	1·67 (0·49–5·75)	0·4080
	No IRS‡	19/564 (3%)	1 (ref)	..
	IRS§	24/604 (4%)	1·55 (0·36–6·58)	0·5468
	Interaction coefficient	..	0·63 (0·12–3·43)	0·5881
Individual group comparison				
	Standard LLIN	7/276 (3%)	1 (ref)	..
	PBO LLIN	12/288 (4%)	1·67 (0·49–5·75)	0·4080
	Standard LLIN plus IRS	12/310 (4%)	1·55 (0·36–6·58)	0·5468
	PBO LLIN plus IRS	12/294 (4%)	1·64 (0·47–5·65)	0·4287

ORs for the factorial analysis compared the two main intervention effects (no PBO LLIN *vs* PBO LLIN, and no IRS *vs* IRS) and their interaction, and compared each of the intervention to the standard LLIN in the individual group analysis. OR was unadjusted for baseline anaemia prevalence. Prevalence of moderate-to-severe anaemia reported in children younger than 5 years with haemoglobin concentrations <8 g/dL. OR=odds ratio. LLIN=long-lasting insecticidal net. PBO=piperonyl butoxide. IRS=indoor residual spraying. ITT=intention to treat.

* Standard LLIN and standard LLIN plus IRS.

† PBO LLIN and PBO LLIN plus IRS.

‡ Standard LLIN and PBO LLIN.

§ Standard LLIN plus IRS and PBO LLIN plus IRS.

A total of 16 371 vector mosquitoes were collected in 5756 indoor light-trap collections over the 2 years. In the first year, vector densities, sporozoite rates, and entomological inoculation rates were lower in the PBO long-lasting insecticidal net groups than in the standard long-lasting insecticidal net groups (table 4), but only entomological inoculation rate was significantly lower in the indoor residual spraying groups than in the non-indoor residual spraying groups. In the second year, the entomological inoculation rate in the PBO long-lasting insecticidal groups remained lower than in the standard long-lasting insecticidal net groups but the effect of indoor residual spraying on entomological inoculation rate had largely diminished by this time compared with the entomological inoculation rates of 2015.

**Table 4:** Entomological outcomes by intervention (PBO LLIN *vs* no PBO LLIN, and IRS *vs* no IRS) in 2015 and 2016

	**Vector density per night per household**				**Sporozoite rate**			EIR per month per household*			
	N	Mean (SD)	DR (95% CI)	p value	n/N (%)	OR (95% CI)	p value	N	Mean (SD)	DR (95% CI)	p value
**Year 1: 2015**											
No PBO LLIN†	896	2·61 (8·97)	1 (ref)	..	20/952 (2%)	1 (ref)	..	862	0·90 (5·42)	1 (ref)	..
PBO LLIN‡	961	1·85 (7·12)	0·33 (0·16–0·69)	0·0038	2/648 (<1%)	0·25 (0·07–0·88)	0·0317	911	0·13 (2·.07)	0·13 (0·03–0·53)	0·0055
No IRS§	939	2·34 (8·18)	1 (ref)	..	21/988 (2%)	1 (ref)	..	901	1·01 (5·85)	1 (ref)	..
IRS¶	918	2·09 (7·96)	0·63 (0·27–1·43)	0·2652	1/612 (<1%)	0·15 (0·02–1·02)	0·0519	872	0·25 (0·89)	0·03 (0·00–0·24)	0·0014
Interaction coefficient	..	..	1·35 (0·44–4·18)	0·5940	..	NA	NA	..	..	NA	NA
**Year 2: 2016**											
No PBO LLIN†	1946	3·60 (16·86)	1 (ref)	..	80/2236 (4%)	1 (ref)	..	1793	1·15 (6·53)	1 (ref)	..
PBO LLIN‡	1953	2·68 (11·33)	0·40 (0·20–0·80)	0·0101	27/1931 (1%)	0·38 (0·15–0·92)	0·0331	1845	0·39 (3·91)	0·33 (0·13–0·83)	0·0189
No IRS§	1942	2·82 (9·34)	1 (ref)	..	64/2207 (3%)	1 (ref)	..	1801	1·00 (6·04)	1 (ref)	..
IRS¶	1957	3·46 (18.01)	0·93 (0·47–1·85)	0·8309	43/1960 (2%)	0·81 (0·37–1·78)	0·5890	1837	0·58 (4·87)	0·48 (0·25–0·94)	0·0340
Interaction coefficient	..	..	1·00 (0·36–2·75)	0·9970	..	1·13 (0·35–3·63)	0·8308	..	..	1·38 (0·47–4·08)	0·5532

DR for vector density and EIR and OR for sporozoite rates are adjusted for their respective baseline value. EIR=entomological inoculation rate. DR=density ratio. OR=odds ratio. LLIN=long-lasting insecticidal net. PBO=piperonyl butoxide. IRS=indoor residual spraying. NA=not applicable.

* The mean and DR of the EIR are weighted to account for the proportion of mosquitoes sampled to be tested for sporozoites. Interaction not estimated in year 1 for sporozoite and EIR outcomes, because sporozoite rate was null in the PBO LLIN plus IRS group.

† Standard LLIN and standard LLIN plus IRS.

‡ PBO LLIN and PBO LLIN plus IRS.

§ Standard LLIN and PBO LLIN.

¶ Standard LLIN plus IRS and PBO LLIN plus IRS.

The mortality of mosquitoes exposed to permethrin for resistance determination in the WHO cylinder tests was 8·8% (95% CI 5·3–12·3; n/N=54/613) for *A gambiae* sensu lato and 54·5% (36·8–76·2; n/N=59/108) for *A funestus*. The lethal concentration required to kill 50% of the wild *A gambiae* sensu lato was 38-times higher and of wild *A funestus* was 34-times higher than for the susceptible reference mosquitoes. The *Vgsc* gene mutation was found in all tested *A gambiae* with co-occurrence of Vgsc-1014F and Vgsc-1014S in 22 (9%) of 234 *A gambiae* mosquitoes. No mutation was found in the 247 *A arabiensis* tested.

## Discussion

This trial showed that long-lasting insecticidal nets incorporating the synergist PBO (Olyset Plus) were more effective than the standard pyrethroid long-lasting insecticidal net (Olyset Net) in reducing malaria infection prevalence in an area of high usage of these nets and high pyrethroid resistance in the primary vectors. The additional effect of the PBO long-lasting insecticidal nets on malaria prevalence was evident at the end of the first year with a 44% protective efficacy and at the end of the second year with a 33% protective efficacy compared with the standard long-lasting insecticidal nets. These findings were supported by the entomological outcomes, which showed a significant reduction in malaria transmission, with entomological inoculation rates being reduced by 87% during the first year and 67% during the second year in areas receiving PBO long-lasting insecticidal nets compared with standard long-lasting insecticidal nets. At 9 months, the addition of pirimiphos-methyl indoor residual spraying to the standard long-lasting insecticidal nets provided similar protection against malaria (44% protective efficacy) relative to the standard nets alone, whereas the addition of indoor residual spraying to PBO long-lasting insecticidal nets did not significantly improve protection based on the interaction observed when both indoor spraying and PBO long-lasting insecticidal nets effect were at their strongest. The impact of indoor residual spraying on the entomological inoculation rates was more than 95% in the first year. This effect on malaria transmission occurred shortly after implementation of indoor residual spraying whereas the effect of PBO long-lasting insecticidal nets took longer. This rapid impact of indoor residual spraying is one reason why this intervention is sometimes more favoured than long-lasting insecticidal net distribution during malaria epidemics, although there has been a paucity of evidence to justify this advice.^27^ Our cluster RCT would support this recommendation, provided high indoor residual spraying coverage can be quickly achieved. Residual insecticidal activity of pirimiphos-methyl on the sprayed walls was observed up to 12 months after a single round of spraying. Following the decay in residual activity during the second year when no spraying was done, the effect of indoor residual spraying on entomological inoculation rates diminished and malaria prevalence increased but not to the level observed in the standard long-lasting insecticidal net control group, which had not received indoor residual spraying in year 1. A sustained effect on malaria transmission would require recurrent annual campaigns of indoor residual spraying.

This trial is the first to provide evidence to suggest that incorporation of the synergist PBO to long-lasting insecticidal nets provides improved community protection compared with standard pyrethroid-only nets against malaria transmission by pyrethroid-resistant vector populations. Previous small-scale experimental hut studies of PBO long-lasting insecticidal nets measured entomological outcomes such as mosquito mortality and biting rates. In Benin, these studies showed that Olyset Plus was more effective than standard Olyset Net against pyrethroid-resistant *A gambiae*, both before and after multiple washing of the nets.^12^ In Tanzania where *A gambiae* was still susceptible to pyrethroids, the differential effect between Olyset Plus and standard Olyset Net was less evident.^28^ Parallel studies with a different type of PBO long-lasting insecticidal net (PermaNet 3.0) showed improved outcomes with the unwashed PBO net compared with the standard long-lasting insecticidal net, but in some studies the efficacy was lost after several washes.^29^ Although these small-scale studies^11^, ^12^, ^28^, ^29^ indicate the potential of PBO nets, they could not capture the full effect of this new class of net on transmission, which is only understood at high coverage levels and in community randomised trials because of the additional community protection that arises from the reduction in mosquito life-span and population density, often called the vectorial mass effect.^27^ With the relatively high coverage and usage of long-lasting insecticidal nets achieved in the present trial (77% usage in the first year and 60% in the second year), we were able to observe a mass effect of the PBO nets on transmission, with concomitant reductions in mosquito density, sporozoite rate, and entomological inoculation rates.

Despite the 83% loss in PBO content after 21 months, the PBO and permethrin retained on the net remained highly effective against malaria infection and entomological inoculation rates throughout. By contrast, the loss of residual activity of the single round of indoor residual spraying of Actellic 300CS led to resumption of transmission and to increasing entomological inoculation rates and malaria prevalence in the second year. The PBO nets will be monitored during a third year to assess whether effectiveness is maintained at low PBO content. There was also a 42% loss of permethrin content in Olyset Plus and 22% in Olyset Net over the two years. The differential release rate of permethrin in the two nets has been observed in other studies, and it has been suggested in an earlier WHO review of Olyset Plus that the more effective performance of the PBO net is due to the higher release rate and surface concentration of permethrin in this net compared with Olyset Net.^28^ Although this argument cannot be completely refuted by our data, the *A gambiae* and *A funestus* vectors in the Muleba area are highly resistant to pyrethroid and any difference in surface permethrin between Olyset Plus and Olyset Net in our trial is unlikely to result in differential mortality rate. A study has shown that under household conditions a 20-times increase in the surface content of permethrin on hand-treated nets causes no increase in mortality to free-flying pyrethroid-resistant *A gambiae*.^30^ Furthermore, synergy tests with PBO showed that pyrethroid-resistant *A gambiae* from our study area are killed by a permethrin concentration they would normally survive if it were not mixed with PBO.^19^

The more effective performance of PBO long-lasting insecticidal nets compared with standard long-lasting insecticidal nets in reducing the prevalence of malaria infection, together with no change in prevalence following the initial distribution and high usage of standard nets, suggests that insecticide resistance of the magnitude reported is compromising the effectiveness of standard pyrethroid nets in northwest Tanzania. A recent study in neighbouring Uganda reported no change in incidence of malaria before and after the distribution of standard long-lasting insecticidal nets.^10^ Other studies have reported the failure of these nets to reduce entomological indicators after the standard long-lasting insecticidal nets developed holes.^5^, ^6^ From our study design, it is not clear whether the standard nets still provide some degree of protection. Although a previous study in Muleba done in 2012 showed that users of standard long-lasting insecticidal nets were slightly better protected (OR 0·83) against malaria infection prevalence than non-users of nets,^31^ this finding should be contrasted with the much larger effect of PBO long-lasting insecticidal nets versus standard nets (OR 0·37) in the present study. In areas with more moderate levels of pyrethroid resistance, standard nets still provide personal protection. A study in Malawi, for example, showed that standard nets reduced malaria incidence by 30% in children in an area where pyrethroid-resistant *A funestus* was the main vector.^8^ In Kenya, the use of standard nets provided 45% protection against the incidence of malaria infection as compared with those not using long-lasting insecticidal nets, but incidence still remained high in net users.^9^ The strength or intensity of resistance in the local primary vector species might be the factor defining the level of protection to be derived from standard long-lasting insecticidal nets.

Our study provides further insight into the question of whether indoor residual spraying and long-lasting insecticidal nets should be combined to accelerate the control of malaria. In a previous cluster RCT in Muleba, where conditions of high pyrethroid resistance and moderate usage of long-lasting insecticidal nets (50%), indoor residual spraying with the carbamate bendiocarb provided an added benefit (OR 0·43).^18^ In the present study, a single round of indoor residual spraying with the long-lasting pirimiphos-methyl capsule suspension in combination with standard nets was sufficient to give long-term additional protection over two transmission seasons (OR 0·33), whereas the bendiocarb required two rounds to achieve an effect of similar size, owing to its shorter residual activity on walls.

The combination of indoor residual spraying of pirimiphos-methyl and PBO long-last insecticidal nets have been suggested to be antagonistic.^13^ This concern arose because pirimiphos-methyl requires oxidation by cytochrome P450 enzymes within the mosquito before it becomes toxic. Uptake of PBO from previous contact with Olyset Plus nets might potentially inhibit this activation process. Although the present cluster RCT neither confirmed nor disproved any antagonistic effect, it showed there was limited benefit to be gained from adding this indoor residual spraying product to PBO nets. Whether another indoor residual spraying insecticide, which does not require activation by cytochrome P450s, would prove an effective partner to PBO long-lasting insecticidal nets is not known. The present cluster RCT also implies that where indoor spraying with pirimiphos-methyl is being applied annually, the substitution of PBO nets for standard nets would provide little or no additional benefit. Considering the focal coverage of indoor residual spraying compared with the much wider coverage of long-lasting insecticidal nets in Africa, an important question from a public health standpoint is which strategy should be adopted in areas where standard long-lasting insecticidal nets might be losing effectiveness because of high intensity of pyrethroid resistance in the local vector? The substitution of PBO long-lasting insecticidal nets in such areas would provide a substantial benefit, similar to that which annual indoor residual spraying campaigns might provide.

This trial has several potential limitations. Buffer areas of 300 m were small compared with what has been used in other trials,^17^, ^18^ which might not have totally prevented contamination. However, any spill-over would have lessened rather than increased the effect size between intervention groups. Additionally, the community was not masked to the indoor residual spraying allocation, which might have led to reduced child attendance at clinic sessions. However, such bias has not been observed and attendance was similar across all intervention groups. Furthermore, we used vector density in CDC light trap collections as a proxy to estimate entomological inoculation rate, rather than vector biting rate in human landing catches. The light trap approach is becoming more common in trials for pragmatic and ethical reasons; and although it could have led to error in the estimation of transmission intensity, it would not have affected the relative difference in entomological inoculation rates observed between the study groups. Finally, our trial was not powered to detect interactions.

In conclusion, this trial shows the residual efficacy of indoor residual spraying with pirimiphos-methyl for malaria control of over 1 year, and provides strong evidence for increasing the coverage of PBO long-lasting insecticidal nets over standard long-lasting insecticidal nets of pyrethroid to meet the increasing challenge of pyrethroid resistance and to improve personal and community protection from malaria, particularly in areas of intense pyrethroid resistance. As a consequence of the trial, WHO has made this policy recommendation.^32^

## References

[1] WHO (2016). World malaria report 2016.

[2] Ranson H, N'Guessan R, Lines J, Moiroux N, Nkuni Z, Corbel V (2011). Pyrethroid resistance in African anopheline mosquitoes: what are the implications for malaria control?. Trends Parasitol.

[3] Barnes KI, Durrheim DN, Little F (2005). Effect of artemether-lumefantrine policy and improved vector control on malaria burden in KwaZulu-Natal, South Africa. PLoS Med.

[4] Kleinschmidt I, Mnzava AP, Kafy HT (2015). Design of a study to determine the impact of insecticide resistance on malaria vector control: a multi-country investigation. Malar J.

[5] Asidi A, N'Guessan R, Akogbeto M, Curtis C, Rowland M (2012). Loss of household protection from use of insecticide-treated nets against pyrethroid-resistant mosquitoes, Benin. Emerg Infect Dis.

[6] Ochomo EO, Bayoh NM, Walker ED (2013). The efficacy of long-lasting nets with declining physical integrity may be compromised in areas with high levels of pyrethroid resistance. Malar J.

[7] Viana M, Hughes A, Matthiopoulos J, Ranson H, Ferguson HM (2016). Delayed mortality effects cut the malaria transmission potential of insecticide-resistant mosquitoes. Proc Natl Acad Sci USA.

[8] Lindblade KA, Mwandama D, Mzilahowa T (2015). A cohort study of the effectiveness of insecticide-treated bed nets to prevent malaria in an area of moderate pyrethroid resistance, Malawi. Malar J.

[9] Ochomo E, Chahilu M, Cook J (2017). Insecticide-treated nets and protection against insecticide-resistant malaria vectors in western Kenya. Emerg Infect Dis.

[10] Katureebe A, Zinszer K, Arinaitwe E (2016). Measures of malaria burden after long-lasting insecticidal net distribution and indoor residual spraying at three sites in Uganda: a prospective observational study. PLoS Med.

[11] Corbel V, Chabi J, Dabire RK (2010). Field efficacy of a new mosaic long-lasting mosquito net (PermaNet 3.0) against pyrethroid-resistant malaria vectors: a multi centre study in western and central Africa. Malar J.

[12] Pennetier C, Bouraima A, Chandre F (2013). Efficacy of Olyset Plus, a new long-lasting insecticidal net incorporating permethrin and piperonyl-butoxide against multi-resistant malaria vectors. PLoS One.

[13] WHO (2015). Conditions for use of long-lasting insecticidal nets treated with a pyrethroid and piperonyl butoxide.

[14] Rowland M, Boko P, Odjo A, Asidi A, Akogbeto M, N'Guessan R (2013). A new long-lasting indoor residual formulation of the organophosphate insecticide pirimiphos methyl for prolonged control of pyrethroid-resistant mosquitoes: an experimental hut trial in Benin. PLoS One.

[15] Oxborough RM (2016). Trends in US President's Malaria Initiative-funded indoor residual spray coverage and insecticide choice in sub-Saharan Africa (2008–2015): urgent need for affordable, long-lasting insecticides. Malar J.

[16] Corbel V, Akogbeto M, Damien GB (2012). Combination of malaria vector control interventions in pyrethroid resistance area in Benin: a cluster randomised controlled trial. Lancet Infect Dis.

[17] Pinder M, Jawara M, Jarju LB (2015). Efficacy of indoor residual spraying with dichlorodiphenyltrichloroethane against malaria in Gambian communities with high usage of long-lasting insecticidal mosquito nets: a cluster-randomised controlled trial. Lancet.

[18] West PA, Protopopoff N, Wright A (2014). Indoor residual spraying in combination with insecticide-treated nets compared to insecticide-treated nets alone for protection against malaria: a cluster randomised trial in Tanzania. PLoS Med.

[19] Matowo J, Kitau J, Kaaya R (2015). Trends in the selection of insecticide resistance in *Anopheles gambiae* s.l. mosquitoes in northwest Tanzania during a community randomized trial of longlasting insecticidal nets and indoor residual spraying. Med Vet Entomol.

[20] Hawley WA, Phillips-Howard PA, ter Kuile FO (2003). Community-wide effects of permethrin-treated bed nets on child mortality and malaria morbidity in western Kenya. Am J Trop Med Hyg.

[21] Gillies MT, Coetzee M (1987). A supplement to the Anophelinae of Africa south of the Sahara (Afrotropical region).

[22] Wirtz RA, Zavala F, Charoenvit Y (1987). Comparative testing of monoclonal antibodies against *Plasmodium falciparum* sporozoites for ELISA development. Bull World Health Organ.

[23] Bass C, Williamson MS, Field LM (2008). Development of a multiplex real-time PCR assay for identification of members of the *Anopheles gambiae* species complex. Acta Trop.

[24] Bass C, Nikou D, Donnelly MJ (2007). Detection of knockdown resistance (*kdr*) mutations in *Anopheles gambiae*: a comparison of two new high-throughput assays with existing methods. Malar J.

[25] Hayes RJ, Moulton LH (2009). Cluster randomised trials.

[26] Drakeley C, Schellenberg D, Kihonda J (2003). An estimation of the entomological inoculation rate for Ifakara: a semi-urban area in a region of intense malaria transmission in Tanzania. Trop Med Int Health.

[27] Pluess B, Tanser FC, Lengeler C, Sharp BL (2010). Indoor residual spraying for preventing malaria. Cochrane Database Syst Rev.

[28] WHO (2012). Report of the fifteenth WHOPES working group meeting: WHO/HQ, Geneva, 18–22 June 2012.

[29] WHO (2009). Report of the twelfth WHOPES working group meeting, WHO/HQ, Geneva 8–11 December 2008.

[30] Corbel V, Chandre F, Brengues C (2004). Dosage-dependent effects of permethrin-treated nets on the behaviour of *Anopheles gambiae* and the selection of pyrethroid resistance. Malar J.

[31] West PA, Protopopoff N, Wright A (2015). Enhanced protection against malaria by indoor residual spraying in addition to insecticide treated nets: is it dependent on transmission intensity or net usage?. PLoS One.

[32] WHO (2017). Conditions for deployment of mosquito nets treated with a pyrethroid and piperonyl butoxide.

