# Supplementary material for: Effectiveness of a long-lasting piperonyl butoxide-treated insecticidal net and indoor residual spray interventions, separately and together, against malaria transmitted by pyrethroid-resistant mosquitoes: a cluster, randomised controlled, two-by-two factorial design trial
Source: Lancet. 2018 Apr 21;391(10130):1577–88. doi: 10.1016/S0140-6736(18)30427-6 (PMC5910376; doi:10.1016/S0140-6736(18)30427-6)
Supplement: Supplementary appendix [file mmc1.pdf]

THE LANCET

Supplementary appendix

This appendix formed part of the original submission and has been peer reviewed. We post it as supplied by the authors.

Supplement to: Protopopoff N, Mosha J F, Lukole E, et al. Effectiveness of a long-lasting piperonyl butoxide-treated insecticidal net and indoor residual spray interventions, separately and together, against malaria transmitted by pyrethroid-resistant mosquitoes: a cluster, randomised controlled, two-by-two factorial design trial. *Lancet* 2018; published online April 11. http://dx.doi.org/10.1016/S0140-6736(18)30427-6.

Supplementary appendix

Contents

1 Methods 2

1.1 Study area 2

1.2 Per-protocol and other analyses 3

2 Results 3

2.1 Coverage of IRS and LLIN per arms and per survey 3

2.2 Per-Protocol analysis result 5

2.3 Comparison of each individual intervention arms with control arm (standard LLIN) 6

2.4 Forest plot: Factorial analysis comparing PBO LLIN vs no PBO LLIN and IRS vs no IRS on malaria infection prevalence 7

2.5 Forest plot: Individual arm analysis comparing each intervention arms with standard LLIN on malaria infection prevalence 8

1 Methods

1.1 Study area

We provide the map of the study area representing each cluster with their core and buffer areas and the intervention allocated.

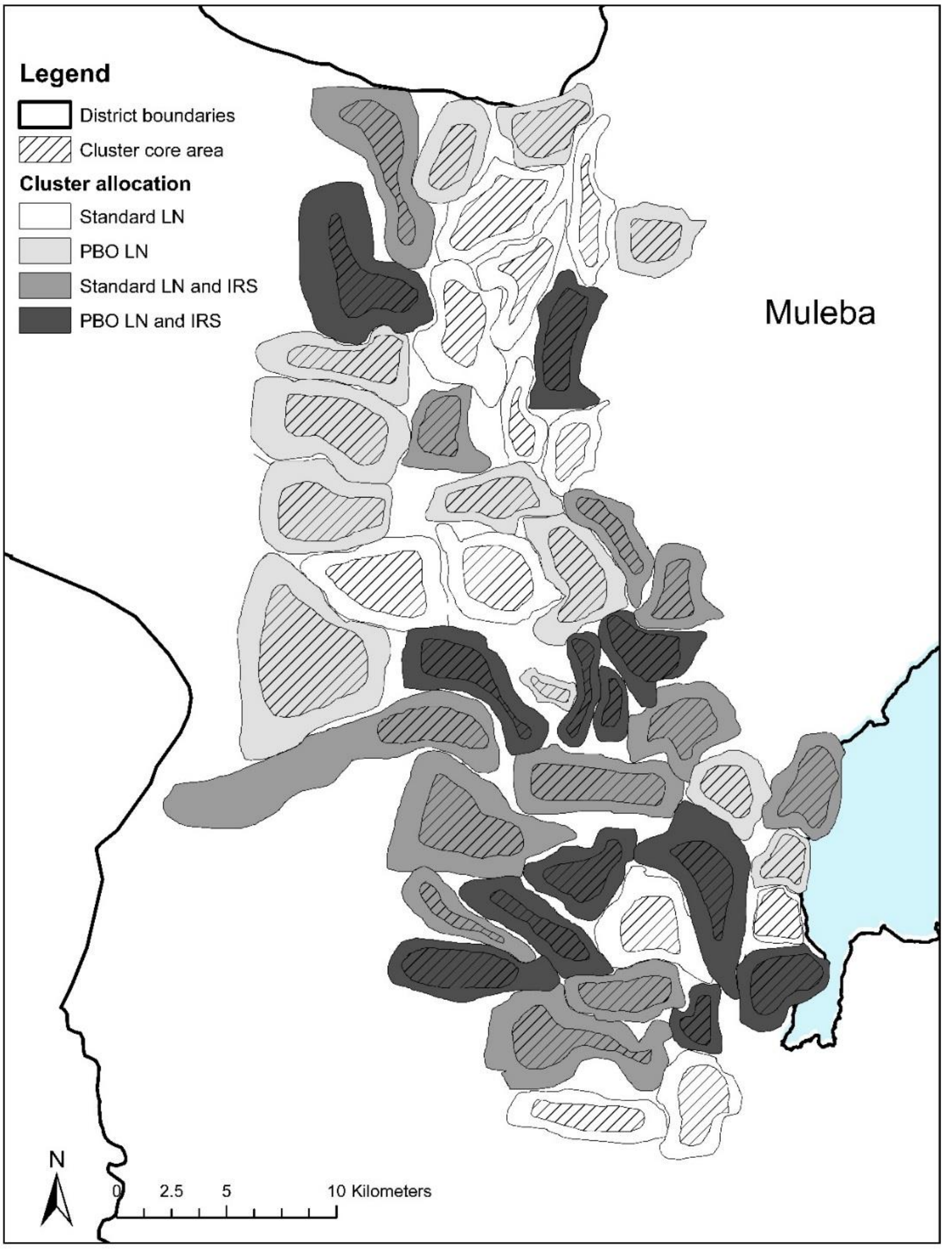

Figure S 1: Map of the study area representing each cluster with their core and buffer areas and the intervention allocated.

1.2 Per-protocol and other analyses

Per-protocol (PP) analysis included only those children who received and used the intended treatment and was restricted to the first-year surveys as it was difficult for householders to recall in the second year whether their house had been sprayed two years earlier. A secondary analysis examined the effect of each intervention arm (1. PBO LLIN alone, 2. Combination standard LLIN & IRS and 3. Combination PBO LLIN & IRS) compared to the reference arm (standard LLIN alone) on entomological inoculation rate, vector density, and sporozoite rate.

2 Results

2.1 Coverage of IRS and LLIN per arms and per survey

Table S 1: Coverage of the interventions by arm.

	Standard LLIN arm	PBO LLIN arm	Standard LLIN & IRS arm	PBO LLIN & IRS arm
Proportion of houses receiving IRS	0.5 (0.1 - 2.0), 2/443	4 (0.5 - 29), 19/425	94 (92 - 96), 408/433	94 (87 - 97), 419/445
Proportion of household owning at least one LLIN (any LLIN*)				
2015 Survey A, 4 months post intervention	97 (88 - 99), 427/442	98 (97 - 99), 419/426	98 (96 - 99), 416/425	97 (93 - 99), 428/439
2015 Survey B, 9 months	97 (93 - 99), 414/425	98 (97 - 99), 398/405	98 (96 - 99), 420/428	98 (95 - 99), 403/412
2016 Survey C, 16 months	96 (94 - 98), 452/470	95 (92 - 96), 449/475	94 (91 - 96), 446/474	93 (90 - 95), 408/439
2016 SurveyD, 21 months	94 (90 - 96), 437/467	92 (87 - 95), 422/460	93 (86 - 97), 418/449	90 (85 - 93), 416/463
Propotion of households with adequate LLIN (any LLIN*) per sleeping place (access)				
2015 Survey A, 4 months	88 (83 -93), 389/440	91 (87 - 94), 387/426	91 (86 - 94), 385/425	89 (82 - 93), 389/439
2015 Survey B, 9 months	91 (88 - 93), 386/424	88 (83 - 92), 358/405	91 (88 - 94), 390/427	87 (82 - 91), 359/411
2016 Survey C, 16 months	80 (75 - 84), 375/470	70 (65 - 74), 332/474	76 (72 - 80), 362/474	71 (63 - 77), 311/439
2016 SurveyD, 21 months	72 (67 - 76), 337/467	69 (62 - 75), 318/460	72 (64 - 79), 323/449	68 (60 - 74), 313/463
Proportion of resident declaring to use a LLIN (any LLIN*) the previous night				
2015 Survey A, 4 months	75 (66 - 82), 1915/2567	78 (73 - 82), 1997/2559	76 (67 - 83), 1893/2493	79 (72 - 85), 2002/2533
2015 Survey B, 9 months	80 (75 - 85), 2012/2510	78 (73 - 82), 1820/2335	76 (70 - 80), 1761/2333	77 (70 - 83), 1941/2522
2016 Survey C, 16 months	68 (59 -76), 1818/2669	58 (51 - 65), 1571/2687	61 (56 - 67), 1629/2652	62 (55 - 69), 1580/2546
2016 SurveyD, 21 months	62 (57 - 67), 1610/2595	54 (47 - 62), 1445/2647	53 (43 - 63), 1368/2570	54 (46 - 62), 1482/2739
Proportion of study LLIN (study LLIN/any LLIN*)				
2015 Survey A, 4 months	92 (89 - 94), 1219/1329	93 (89 - 95), 1241/1337	95 (91 - 97), 1175/1241	94 (91 - 96), 1214/1291
2015 Survey B, 9 months	95 (92 - 97), 1144/1199	94 (91 - 96), 1043/1110	93 (90 - 95), 1042/1122	93 (89 - 96), 1132/1216
2016 Survey C, 16 months	92 (89 - 94), 1034/1126	91 (86 - 94), 981/1081	90 (85 - 94), 1018/1131	90 (84 - 94), 913/1013
2016 SurveyD, 21 months	82 (77 - 85), 844/1025	77 (71 - 82), 785/1021	79 (73 - 83), 789/1004	69 (61 - 76), 771/1122

* Any LN include study LLIN and other LLIN

Results are expressed in proportion with (95%CI) and absolute number n/N. N is total household number for IRS, LLIN ownership and LLIN access and total number of resident for usage.

2.2 Per-Protocol analysis result

Table S 2: Per-protocol analysis Plasmodium infection prevalence and anaemia by intervention (PBO LLIN vs No PBO LLIN and IRS vs No IRS), 4 and 9 months post intervention.

	Plasmodium infection prevalence					Anaemia prevalence under 5				
	n/N	%	OR	95%CI	P-value	n/N	%	OR	95%CI	P-value
**Survey A, 2015, 4 months post intervention**										
No PBO LLIN [1]	618/1316	47·0	1			20/448	4·5	1		
PBO LLIN [2]	546/1329	41·1	0·63	0·36 - 1·13	0·1190	11/483	2·3	0·49	0·15-1·52	0·2105
No IRS [3]	670/1329	50·4	1			19/470	4.0	1		
IRS [4]	494/1316	37·5	0·47	0·27 - 0·83	0·0099	12/461	2·6	0·61	0·21-1·77	0·3579
Interaction coefficient			1·56	0·73-3·33	0·2435			1·01	0·18-5·80	0·9864
**Survey B, 2015, 9 months post intervention**										
No PBO LLIN [1]	535/1223	43·7	1			11/383	2·9	1		
PBO LLIN [2]	341/1202	28·4	0·31	0·18 - 0·53	0·0001	5/399	1·3	0·23	0·06-0·86	0·0292
No IRS [3]	562/1272	44·2	1			13/404	3·2	1		
IRS [4]	314/1153	27·2	0·28	0·16 - 0·47	<0·0001	3/378	0·8	0·08	0·01-0·62	0·0159
Interaction coefficient			3·03	1·46-6·29	0·0037			10·12	0·74-137·92	0·0811

Per-protocol analysis restricted to individuals receiving and using the allocated interventions in each arm in year 2015. Data presented are n/N (%) and 95%CI. OR compared the two-main intervention effect 1/PBO LLIN vs No PBO LLIN and 2/IRS vs no IRS and their interaction. OR unadjusted for baseline plasmodium infection prevalence or anaemia prevalence. Plasmodium infection prevalence is reported for children 6 months to 14 years and prevalence of moderate to severe anaemia in children under 5 years old with haemoglobin level <8g/dl.

[1] Standard LLIN and standard LLIN & IRS arms, [2] PBO LLIN and PBO LLIN & IRS arms, [3] standard LLIN and PBO LLIN arms, [4] standard LLIN & IRS and PBO LLIN & IRS arms.

2.3 Comparison of each individual intervention arms with control arm (standard LLIN)

Table S 3: Mean vector density per night per household, sporozoite rate, and entomological inoculation rate per month per household by study arms during the first year and second year post intervention.

	Vector density per night per household					Sporozoite rate					EIR per month per household				
	N	Mean	DR	95%CI	P-value	n/N	%	OR	95%CI	P-value	N	Mean*	DR*	95%CI	P-value
**Year 1: 2015**															
Standard LN arm	471	2·83	1			19/683	2·8%	1			449	1·76	1		
PBO LN arm	468	1·84	0·33	0·16-0·69	0·0038	2/305	0·7%	0·247	0·07-0·88	0·03171	452	0·26	0·13	0·03-0·53	0·0055
Standard LN & IRS arm	425	2·37	0·63	0·27-1·43	0·2652	1/269	0·4%	0·149	0·02-1·02	0·05194	413	0·05	0·03	0·004-0·24	0·0014
PBO LN & IRS arm	493	1·853	0·28	0·13-0·60	0·0016	0/343	0.0%	-			459	0.00	-		
**Year 2: 2016**															
Standard LN arm	966	3·04	1			50/1268	3·9%	1			881	1·65	1		
PBO LN arm	976	2·6	0·4	0·20-0·80	0·0101	14/939	1·5%	0·38	0·15-0·92	0·0331	920	0·43	0·33	0·13-0·83	0·0189
Standard LN & IRS arm	980	4·15	0·93	0·47-1·85	0·8309	30/968	3·1%	0·81	0·37-1·78	0·589	912	0·78	0·48	0·25-0·94	0·034
PBO LN & IRS arm	977	2·76	0·37	0·22-0·65	0·0008	13/992	1·3%	0·34	0·13-0·90	0·0309	925	0·35	0·22	0·11-0·45	0·0001

*Mean and DR accounting for sampling weight

2.4 Forest plot: Factorial analysis comparing PBO LLIN vs no PBO LLIN and IRS vs no IRS on malaria infection prevalence

Figure S 2: Forest plot for the factorial analysis including the protective efficacy (PE=1-malaria prevalence ratio) between intervention arms and reference arms), the OR and 95%CI, 4, 9, 16 and 21 months post intervention.

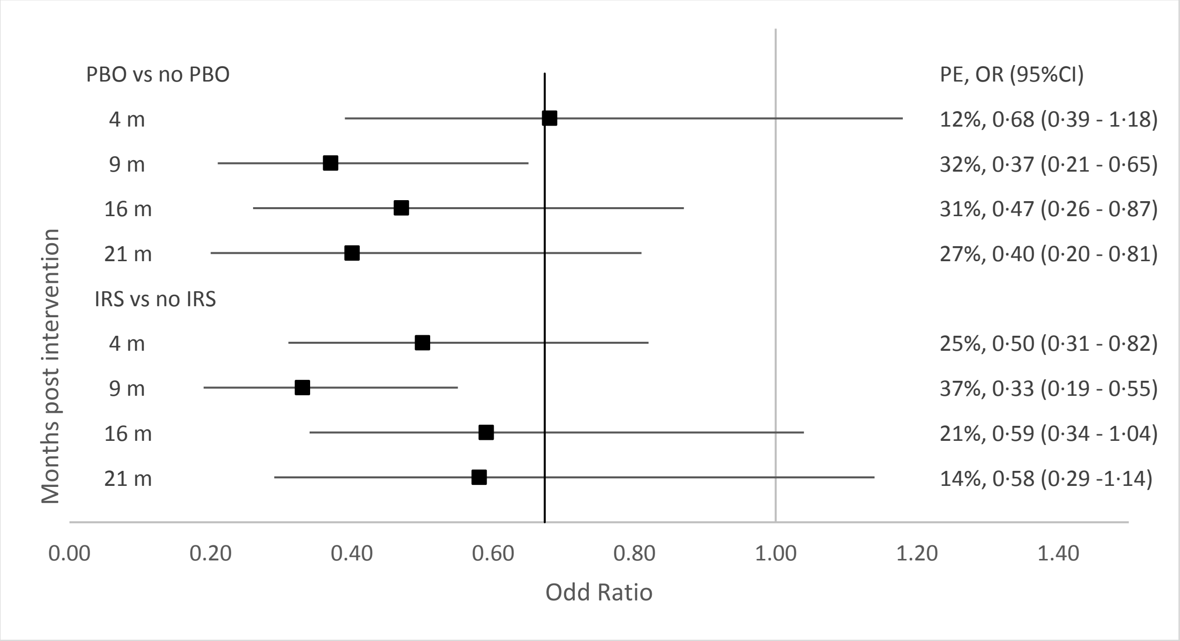

The prevalence ratios of 0.72 (28% reduction) and a baseline prevalence of 20% in the reference arm as pre- specified in the sample size calculation, translates to odds ratio of 0.673. This OR is represented by the vertical black line.

2.5 Forest plot: Individual arm analysis comparing each intervention arms with standard LLIN on malaria infection prevalence

Figure S 3: Forest plot for the comparison of each intervention to the control arm (standard LLIN) including the protective efficacy (PE), the OR and 95%CI, 4, 9, 16 and 21 months post intervention.

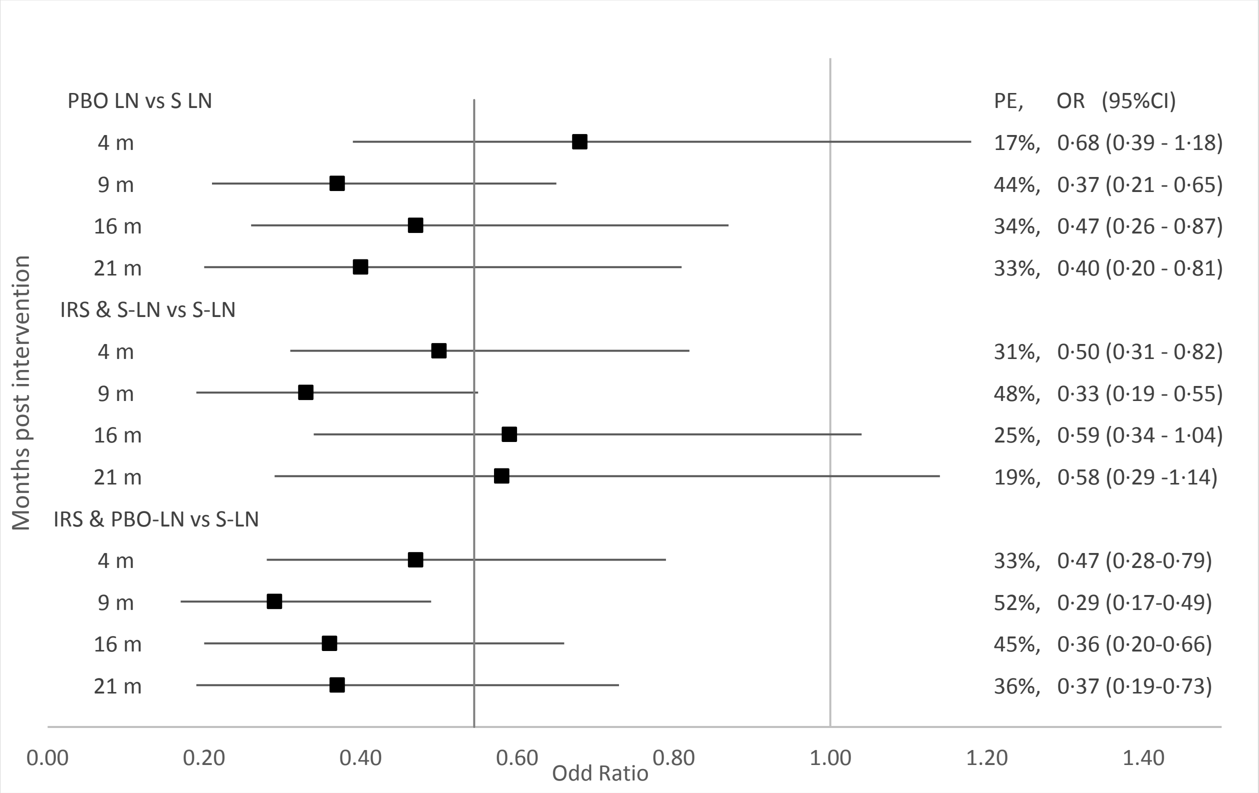

The prevalence ratios of 0.60 (40% reduction) with a baseline prevalence of 20% in the reference arm as pre- specified in the sample size calculation, translates to odds ratio of 0.545. This OR is represented by the vertical black line.
